# Daily fluid balance predicts hospital mortality in critically ill patients receiving continuous renal replacement therapy

**DOI:** 10.1186/cc12371

**Published:** 2013-03-19

**Authors:** M Kashiouris, A Akhoundi, S Chaudhary, V Velagapudi, A Goldberg, K Kashani

**Affiliations:** 1 Mayo Clinic, Rochester, MN, USA

## Introduction

It has been suggested that fluid balance is a biomarker in critically ill patients [1]. There is a paucity of randomized trials examining the effect of daily fluid balance on outcomes in patients on continuous renal replacement therapy (CRRT). The RENAL trial did not find mortality difference with higher CRRT dose [2], but did not investigate the effect of daily fluid balance on patient outcomes. A *post hoc *analysis suggested survival benefit in patients with negative fluid balance [3]. In this study, we hypothesize that daily fluid balance is an independent predictor of mortality in critically ill patients.

## Methods

We conducted a retrospective cohort study in eight ICUs of a tertiary academic center. We constructed a robust clustered linear regression model of daily fluid balance and all-cause hospital mortality among 595 critically ill patients receiving CRRT. We adjusted the model for the Charlson comorbidity score, the daily SOFA scores in the first week after initiation of CRRT as well the type of ICU.

## Results

**Figure 1:**
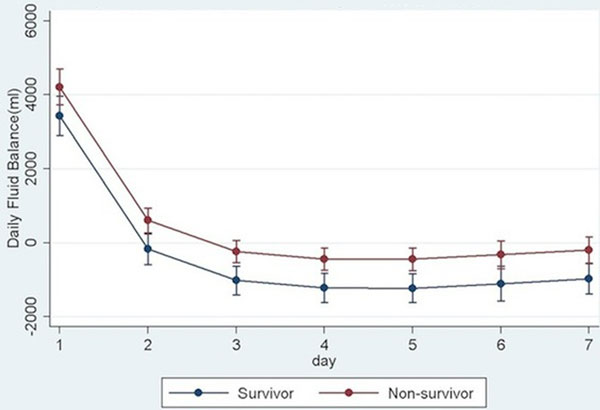
**Daily fluid balance and 95% CIs among survivors and nonsurvivors**.

After adjusting for the type of ICU and the daily severity of illness, patients who died had on average 779 ml higher daily fluid balance compared with patients who survived (*P *0.001, 95% CI = 385 to 1,173 ml, Figure 1). Severity of illness predicted daily fluid accumulation; each additional point of the SOFA score predicted an additional 57 ml of extra daily fluid (*P *= 0.002).

## Conclusion

Among critically ill patients who are receiving CRRT, daily fluid balance is an independent predictor of mortality, even after adjusting for the daily severity of illness and comorbidities.
